# Effects of volume management on free flap perfusion and metabolism in a large animal model study

**DOI:** 10.1038/s41684-024-01410-6

**Published:** 2024-08-09

**Authors:** Daniel G. E. Thiem, Daniel Stephan, Alexander Ziebart, Robert Ruemmler, Julian Riedel, Shankeeth Vinayahalingam, Bilal Al-Nawas, Sebastian Blatt, Peer W. Kämmerer

**Affiliations:** 1 Department of Oral and Maxillofacial Surgery, Facial Plastic Surgery, University Medical Centre of the Johannes Gutenberg-University Mainz, Mainz, Germany; 2 Department of Anaesthesiology, University Medical Centre of the Johannes Gutenberg-University Mainz, Mainz, Germany; 3 Department of Oral and Maxillofacial Surgery, Radboud University Medical Center, Nijmegen, The Netherlands

**Keywords:** Experimental models of disease, Preclinical research, Reconstruction

## Abstract

Free flap failure represents a substantial clinical burden. The role of intraoperative volume management remains controversial, with valid studies lacking. Here, using a large animal model, we investigated the influence of volume management on free flap perfusion and metabolism. Autotransfer of a musculocutaneous gracilis flap was performed on 31 German domestic pigs, with arterial anastomosis and catheterization of the pedicle vein for sequential blood sampling. Flap reperfusion was followed by induction of a hemorrhagic shock with maintenance for 30 min and subsequent circulation stabilization with crystalloid solution, crystalloid solution and catecholamine, autotransfusion or colloidal solution. Flap perfusion and oxygenation were periodically assessed using hyperspectral imaging. Flap metabolism was assessed via periodic blood gas analyses. Hyperspectral imaging revealed no difference in either superficial or deep tissue oxygen saturation, tissue hemoglobin or tissue water content between the test groups at any time point. Blood gas analyses showed that lactate levels were significantly increased in the group that received crystalloid solution and catecholamine, after circulatory stabilization and up to 2 h after. We conclude that, in hemorrhagic shock, volume management impacts acid–base balance in free flaps. Crystalloid solutions with norepinephrine increase lactate levels, yet short-term effects on flap perfusion seem minimal, suggesting that vasopressors are not detrimental.

## Abstract (web summary)

Free flap is widely used in head and neck reconstructive surgery, but fluid management of surgical patients remains controversial. Here, Thiem et al. used a pig model to monitor the effects of volume management on free flap perfusion and metabolism.

## Main

In reconstructive oral and maxillofacial surgery, the transfer of free flaps is a critical and commonly performed method for reconstructing defects in the head and neck region. The success of free microvascular flap transfer is often defined by the generally high (96%) flap survival rate. However, this high success rate is largely based on the good results achieved using less complex flaps (for example, fasciocutaneous radial or ulnar forearm flaps) and does not fully apply to more complex composite or perforator flaps^1–3^. In addition to the complexity of the flap, other factors such as prolonged surgery duration, the need for intraoperative re-anastomosis, anatomically complex flap sites, challenging microanastomoses and arterial or venous thrombosis can contribute to flap revision or early flap failure^4^. Partial or complete flap loss, usually caused by malperfusion (venous > arterial), can lead to a substantial increase in patient morbidity and mortality due to wound healing disorders, necessary second interventions, delayed adjuvant radio- and/or chemotherapy, and prolonged hospital stays^5,6^. Furthermore, these complications impose an additional financial burden on healthcare systems^7^.

To mitigate these risks, close perioperative flap monitoring has been established as the only effective tool for early detection of malperfusion and timely re-exploration. Clinical assessment, despite being subjective and poorly reproducible, is still considered the gold standard for flap monitoring^8^. Hyperspectral imaging (HSI) is a new and relatively unexplored methodological field in medical applications, and our group has demonstrated its successful usability for free flap monitoring in both preclinical animal studies and retrospective and prospective clinical studies^9–14^.

Patient physiology substantially impacts flap viability; however, anesthetic management and postoperative care also directly affect outcomes. Reconstructive procedures using free flaps, typically lasting 6–8 h, involve multiple tissue trauma sites, leading to substantial fluid, blood and heat losses. Unaddressed hypovolemic vasoconstriction and hypothermia can potentially jeopardize flap perfusion and survival. Even with suitable fluid therapy approaches, flap perfusion might decrease by 50% in the initial 6–12 h following surgery. When considering that blood flow is laminar, blood flow factors are influenced by perfusion pressure, viscosity, vessel length and radius. Whereas the latter two parameters cannot be influenced, blood volume and blood pressure can be controlled. To maintain proper perfusion pressure in transplanted flaps, a hyperdynamic circulation featuring high cardiac output, pulse pressure and peripheral vasodilation is essential. Sufficient blood pressure combined with vasodilation facilitates optimal flap perfusion by augmenting regional blood flow, promoting microvascular patency and preserving blood fluidity within microcirculation.

Numerous clinical associations have been established through retrospective analysis of clinical data or prospective evaluation of uncontrolled trial outcomes. Although the inclusion of a heterogeneous patient population reflects real-world scenarios, the substantial variability observed in the patient cohort makes generalized conclusions challenging. In certain instances, the ethical considerations surrounding a research question render a controlled and randomized patient study infeasible. In such scenarios, animal experimentation serves as a commonly employed alternative. Therefore, we decided to use pigs (*Sus scrofa*) as a large animal model for our study given that they are increasingly adopted in medical research due to their similarities with humans in terms of anatomy, physiology and metabolism^15^. Additionally, pigs have a pivotal role in translational medicine, particularly in organ transplantation^16,17^.

The possibility of influencing flap perfusion through the use of vasoconstrictors has been a source of controversy and discussion for a considerable period of time. Therefore, the objective of this study was to provide insights into the effects of hemorrhagic shock and volume therapy on free flap perfusion and tissue oxygenation, with implications for future research in this area.

## Results

A total of 37 animals were used, of which 6 animals died early due to cardiac arrhythmia and had to be excluded. The remaining 31 animals were randomly assigned to group Sterofundin only (STERO; *n* = 7), Sterofundin + norepinephrine (STERO+; *n* = 8), autotranfusion (*n* = 8; AUTO) or Gelafundin (GELA; *n* = 8). A graphical abstract representing the full experimental setup can be found in Supplementary Fig. 1.

### Hyperspectral perfusion imaging

#### Tissue oxygen saturation

**Fig. 1:**
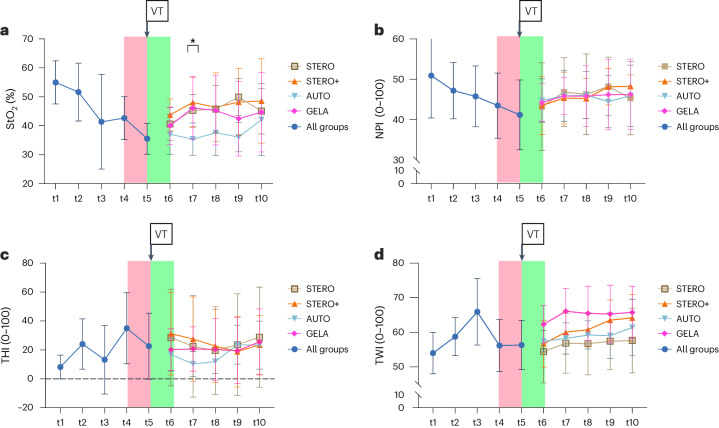
Time-dependent changes in hyperspectral parameters across different groups. **a**–**d**, Line charts (mean ± s.d.) display the hyperspectral parameters StO_2_ (**a**), NPI (**b**), THI (**c**) and TWI (**d**) at various measurement time points from t1 to t10, comparing the different groups STERO (*n* = 7), STERO+ (*n* = 8), AUTO (*n* = 8) and GELA (*n* = 8) starting from t6. The red area indicates the period from flap reperfusion to the state of hemorrhagic shock. The green area marks the period of circulatory stabilization (start volume therapy, VT). Statistical significance with *P* < 0.05 (*) was assessed using one-way ANOVA and post-hoc LSD analyses. t1, preOP; t2, transplant (Tx) preparation; t3, pre-Tx reperfusion; t4, post-Tx reperfusion; t5, shock; t6, post resuscitation; t7 = t6 + 1 h; t8 = t6 + 2 h; t9 = t6 + 3 h; t10 = t6 + 4 h.

The mean tissue oxygen saturation (StO_2_) values ranged from 35.24 ± 5.3% to 53.98 ± 7.1%, with the lowest means observed during hemorrhagic shock (t5) and the highest means observed at preoperative baseline (t1) (Fig. 1a). The results revealed no significant difference in StO_2_ levels between groups after circulation stabilization (t6, *P* = 0.270; t8, *P* = 0.411; t9, *P* = 0.087; and t10, *P* = 0.830; one-way analysis of variance (ANOVA) and least significant difference (LSD) post-hoc analyses). However, there was a significant difference in StO_2_ levels between groups at t7, 1 h after circulation stabilization (*P* = 0.049; one-way ANOVA) referring to the comparison of AUTO and STERO (*P* = 0.048), STERO+ (*P* = 0.009) and GELA (*P* = 0.027, post-hoc LSD analyses) (Fig. 1a).

#### Near-infrared perfusion index

Near-infrared perfusion index (NPI) results demonstrated the highest means at preoperative baseline (t1) with 50.9 ± 10.1, a gradual decrease over time (t2 to t4) and the lowest means during hemorrhagic shock (t5) with 41.2 ± 8.2. After circulatory stabilization, NPI continuously increased until t10, reaching 46.9 ± 7.1 (Fig. 1b). Intergroup comparison of NPI between STERO, STERO+, AUTO and GELA groups revealed no difference from t6 to t10 (*P* > 0.5, one-way ANOVA; Fig. 1b).

#### Tissue hemoglobin index

Tissue hemoglobin index (THI) results demonstrated the lowest means at preoperative baseline (t1) with 7.9 ± 8.1 and the highest means after flap reperfusion (t4) with 36.05 ± 24.8. Subsequently, THI demonstrated unspecific changes until t10, characterized by sequential fluctuations in value (Fig. 1c). There was no significant difference in THI between STERO, STERO+, AUTO and GELA groups at any time point (*P* > 0.5, one-way ANOVA).

#### Tissue water index

Tissue water index (TWI) increased from baseline (t1; 54.05 ± 5.9) to pre-reperfusion (t3; 65.94 ± 9.6), subsequently dropped at t4 (55.9 ± 7.4) and during the hemorrhagic shock (t5; 56.4 ± 7.0), and then gradually increased until t10, reaching 62.61 ± 7.9. There was no significant difference in TWI between STERO, STERO+, AUTO and GELA groups at any time point from t6 (*P* > 0.08, one-way ANOVA; Fig. 1d).

### Blood gas analysis

The values in the venous blood gas analysis (BGA) correspond to the time points following flap reperfusion, and notably post-arterial anastomosis (t4).

#### pO_2_ and pCO_2_

**Fig. 2:**
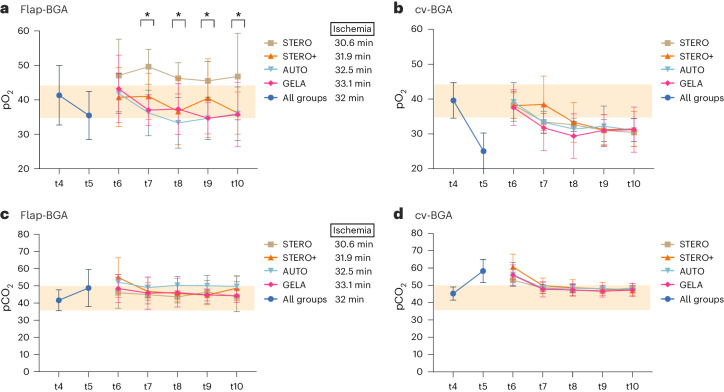
Venous BGA of pO_2_ and pCO_2_. **a**–**d**, Line charts (mean ± s.d.) show pO_2_ (**a** and **b**) and pCO_2_ concentration (**c** and **d**) in venous BGA obtained from the flap (flap-BGA, **a** and **c**) and the central venous circulation (cv-BGA, **b** and **d**) at the measurement time points t4 to t10 comparing the groups STERO (*n* = 7), STERO+ (*n* = 8), AUTO (*n* = 8) and GELA (*n* = 8) starting from t6. Average flap ischemia durations (in minutes) are indicated next to the names of each group. Statistical significance with *P* < 0.05 (*) was assessed using one-way ANOVA and post-hoc LSD analyses. t4, post-transplant (Tx) reperfusion; t5, shock; t6, post resuscitation; t7 = t6 + 1 h; t8 = t6 + 2 h; t9 = t6 + 3 h; t10 = t6 + 4 h.

After reperfusion of the flap (t4), there was no significant difference in pO_2_ levels between flap-BGA (pO_2_^(flap-BGA)^) and central venous BGA (pO_2_^(cv-BGA)^) (*P* = 0.057, Wilcoxon signed-rank test). During the hemorrhagic shock (t5), pO_2_ levels dropped, with a significantly higher decrease in pO_2_^(cv-BGA)^ compared to pO_2_^flap-BGA^ (*P* < 0.001, Wilcoxon signed-rank test). After circulatory stabilization (t6), pO_2_^flap-BGA^ did not differ between the different groups (STERO, STERO+, AUTO and GELA; *P* = 0.57, one-way ANOVA). From t7 to t10, pO_2_^(flap-BGA)^ was significantly increased in the STERO group compared to STERO+, AUTO and GELA (*P* < 0.05, one-way ANOVA) (Fig. 2a,b).

pCO_2_^(cv-BGA)^ levels were significantly higher than pCO_2_^(flap-BGA)^ after both flap reperfusion (t4; *P* < 0.001, Wilcoxon signed-rank test) and during hemorrhagic shock (t5; *P* < 0.001, Wilcoxon signed-rank test). No significant differences in pCO_2_ levels were noted between STERO, STERO+, AUTO and GELA groups at any other time point for pCO_2_^flap-BGA^ (*P* > 0.2, one-way ANOVA; Fig. 2c,d).

#### pH and lactate

**Fig. 3:**
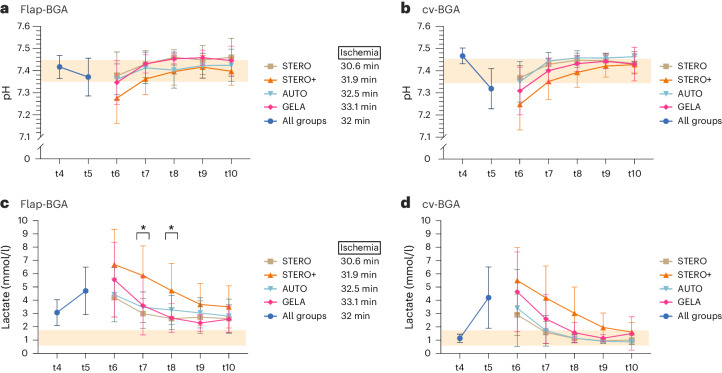
Analysis of pH and lactate levels in flap and central venous blood samples. **a**–**d**, Line charts (mean ± s.d.) show pH (**a** and **b**) and lactate (**c** and **d**) in venous blood samples obtained from the flap (flap-BGA, **a** and **c**) and the central venous circulation (cv-BGA, **b** and **d**) at the measurement time points t4 to t10 comparing the groups STERO (*n* = 7), STERO+ (*n* = 8), AUTO (*n* = 8) and GELA (*n* = 8) starting from t6. *P < 0.05. Differences between groups were assessed using one-way ANOVA and post-hoc LSD analyses. t4, post-transplant (Tx) reperfusion; t5, shock; t6, post resuscitation; t7 = t6 + 1 h; t8 = t6 + 2 h; t9 = t6 + 3 h; t10 = t6 + 4 h.

After flap re-prefusion (t4), pH^flap-BGA^ was significantly lower compared to pH^cv-BGA^ (*P* < 0.001, Wilcoxon signed-rank test), whereas pH^cv-BGA^ was significantly lower compared to pH^flap-BGA^ at t5 (*P* < 0.001, Wilcoxon test; Fig. 3a,b). No significant variation in pH^flap-BGA^ was observed between the STERO, STERO+, AUTO and GELA groups during the time of circulatory stabilization (t6) and at subsequent measurement time points (t7 to t10) (*P* > 0.05, one-way ANOVA; Fig. 3a).

Statistically significant differences were detected between lactate^flap-BGA^ and lactate^cv-BGA^ after flap reperfusion (t4) as well as during hemorrhagic shock (t5) with higher values in flap-BGA (*P* < 0.002, Wilcoxon signed-rank test; Fig. 3c,d). The mean lactate^flap-BGA^ levels decreased from 5.31 mmol/l at t6 to 2.8 mmol/l at t10, while mean lactate^cv-BGA^ levels decreased from 4.21 mmol/l at t6 to 1.29 mmol/l at t9, and then slightly increased to 1.29 mmol/l at t10 (Fig. 3c,d). One (t7) and two hours (t8) after achieving resuscitation, the STERO+ group showed significantly higher lactate^flap-BGA^ levels compared to the STERO (*P* = 0.006 and *P* = 0.006, respectively), AUTO (*P* = 0.023 and *P* = 0.006, respectively) and GELA (*P* = 0.020 and *P* = 0.006, respectively) groups (post-hoc LSD analyses; Fig. 3c).

#### Electrolytes

**Fig. 4:**
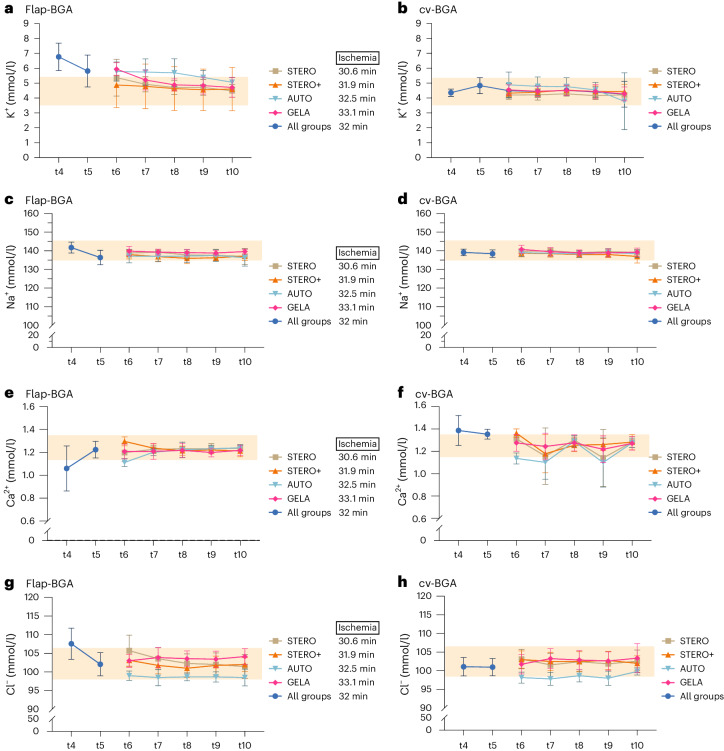
Electrolyte concentrations in venous blood samples. **a**–**h**, Line charts (mean ± s.d.) show the concentration of various electrolytes (K^+^, Na^+^, Ca^2+^ and Cl^−^) in venous blood samples obtained from the flap (flap-BGA; **a**,**c**,**e**,**g**) and the central venous circulation (cv-BGA; **b**,**d**,**f**,**h**) at measurement time points t4 to t10 comparing the groups STERO (*n* = 7), STERO+ (*n* = 8), AUTO (*n* = 8) and GELA (*n* = 8) starting from t6. *P < 0.05 (one-way ANOVA and post-hoc LSD analyses). t4, post-transplant (Tx) reperfusion; t5, shock; t6, post resuscitation; t7 = t6 + 1 h; t8 = t6 + 2 h; t9 = t6 + 3 h; t10 = t6 + 4 h.

Following flap reperfusion (t4) as well as during hemorrhagic shock (t5), K^+ flap-BGA^ levels were significantly higher than K^+ cv-BGA^ levels (*P* < 0.001, Wilcoxon signed-rank test; Fig. 4a,b). The mean K^+ flap-BGA^ levels decreased from 5.5 ± 1.1 mmol/l at t6 to 4.7 ± 0.8 mmol/l at t10, while mean K^+ cv-BGA^ decreased from 4.5 ± 0.5 mmol/l at t6 to 4.2 ± 0.9 mmol/l at t10. K^+^ values differed significantly between flap-BGA and cv-BGA at all time points (t6 to t10) (*P* < 0.001, Wilcoxon signed-rank test; Fig. 4a,b). By contrast, K^+ flap-BGA^ levels did not differ between the STERO, STERO+, AUTO and GELA groups (*P* > 0.05, post-hoc LSD analyses; Fig. 4a).

After reperfusion (t4), Na^+ flap-BGA^ levels were significantly higher than Na^+ cv-BGA^ (*P* < 0.001, Wilcoxon signed-rank test), whereas Na^+^ levels were higher in cv-BGA compared to flap-BGA during hemorrhagic shock (t5) (*P* = 0.001, Wilcoxon signed-rank test). The mean Na^+ flap-BGA^ levels slightly decreased from 138.0 ± 3.1 mmol/l at t6 to 137.4 ± 3.3 mmol/l at t10. Mean Na^+ cv-BGA^ levels slightly decreased from 139.3 ± 2.1 mmol/l at t6 to 138.4 ± 2.4 mmol/l at t10. Na^+^ values differed significantly between flap-BGA and cv-BGA at all time points (*P* ≤ 0.002, Wilcoxon signed-rank test) except at t10 (*P* = 0.12; Fig. 4c,d). By contrast, Na^+ flap-BGA^ levels did not differ between STERO, STERO+, AUTO and GELA groups (*P* > 0.05; Fig. 4c).

After flap reperfusion (t4) and during hemorrhagic shock (t5), Ca^2+ flap-BGA^ levels were significantly lower than Ca^2+ cv-BGA^ levels (*P* < 0.001; Wilcoxon signed-rank test). Mean Ca^2+ flap-BGA^ levels slightly decreased from 1.22 ± 0.07 mmol/l at t5 to 1.19 ± 0.22 mmol/l at t10, while mean Ca^2+ cv-BGA^ levels remained relatively stable over time, ranging from 1.35 ± 0.04 mmol/l at t5 to 1.2791 ± 0.05 mmol/l at t10 (Fig. 4e,f). There were significant differences between flap-BGA and cv-BGA values at the time of circuit stabilization (t6) as well as 2 h (t8) and 4 h after (t10) (*P* < 0.001, Wilcoxon signed-rank test). At the point of hemodynamic stabilization (t6), Ca^2+ flap-BGA^ levels differed significantly among all groups (*P* = 0.001, one-way ANOVA) except between STERO and GELA (*P* = 0.796, post-hoc LSD analysis). The highest Ca^2+^ concentration was observed in the STERO+ group at 1.29 mmol/l, followed by GELA at 1.207 mmol/l, STERO at 1.19 mmol/l and AUTO at 1.11 mmol/l. At subsequent measurement points (t7 to t10), the experimental groups showed no significant differences (*P* > 0.29, one-way ANOVA).

After flap reperfusion (t4) and during hemorrhagic shock (t5), Cl^− flap-BGA^ levels were significantly higher than Cl^− cv-BGA^ levels (*P* < 0.001; Wilcoxon signed-rank test). Mean Cl^− flap-BGA^ levels slightly decreased from 107.56 ± 4.17 mmol/l at t4 to 101.52 ± 2.91 mmol/l at t10, while mean Cl^− cv-BGA^ levels remained relatively stable over time, ranging from 101.09 ± 2.47 mmol/l at t4 to 102.12 ± 2.85 mmol/l at t10 (Fig. 4g,h). There were significant differences between the Cl^− flap-BGA^ and Cl^− cv- BGA^ values at the time of circulation stabilization (t6) (*P* = 0.045, Wilcoxon signed-rank test) and nonsignificant differences from t7 to t10 (*P* > 0.11, Wilcoxon signed-rank test). From hemodynamic stabilization (t6) to 4 h later (t10), Cl^− flap-BGA^ levels significantly differed among the groups at all time points (*P* < 0.013). Throughout this period, the AUTO group consistently showed significantly lower Cl^−^ levels compared to the other groups at t7 (*P* = 0.004), t8 (*P* = 0.004), t9 (*P* = 0.013) and t10 (*P* = 0.003, post-hoc LSD analyses).

## Discussion

The free flap model showcased in this research draws inspiration from a method recently introduced by Gonzalez-Garcia et al.^18^. We have incorporated several novel modifications not previously outlined, such as the combination of hemorrhagic shock with a free flap transfer, choosing the axillary region as the recipient site, and introducing pedicle catheterization for consistent venous blood sampling exclusive to the flap. Animals were consistent in weight, age and race throughout the whole experiment, and hereby variability in vessel characteristics and vessel diameter was minimized. Although no specific measurements of vessel diameters were performed, this experimental model closely mimics the anatomical features and suturing techniques of pedicle vessels observed in clinical scenarios. Notably, despite potential variations in vessel size across flap types and recipient sites for both humans and animals, the impact of pedicle vessel diameter on the presented results is negligible since the uniformity in the weight, age and race of the selected animals reduces variability in vessel characteristics, making any differences in vessel diameter minimal. Second, the microsurgical techniques used, such as anastomosis, are highly effective across a range of vessel sizes, ensuring vessel patency regardless of minor diameter variations. Third, vascular healing processes are resilient to small differences in vessel diameter, with endothelial cell function and smooth muscle activity maintaining normal responses when surgical techniques are precise. Therefore, the primary focus on the effectiveness of the techniques and outcomes is maintained, making the exact diameter of the pedicle vessels a minor factor in the overall results.

BGA, a prevalent diagnostic approach, assesses the partial pressures of gases in blood and acid–base content, aiding in the interpretation of respiratory, circulatory and metabolic disorders^19^. Although venous BGA provides less accurate values compared to arterial BGA, it provides valuable information in the context of the aforementioned dysregulations^20^. In the current study, dysregulated metabolic processes, tissue damage (as measured by lactate levels) and gas exchange disturbances in the flap itself were evaluated by comparing flap-specific venous blood gas and central venous blood gas.

### Fluid therapy for circulatory stabilization

In our research, aside from elevated lactate levels in animals administered with the crystalloid solution and norepinephrine (STERO+), there were no discernible differences in the metabolism and perfusion of the free gracilis flap across the STERO, STERO+, AUTO and GELA groups. The application of vasoconstrictors in free flap surgery to manage hypotension remains contentious. While animal studies have demonstrated vasoconstriction in flap microcirculation after administration of vasopressors^21^, the use of intraoperative vasoconstrictor does not impact flap outcomes in human head and neck cancer surgery or other microsurgical procedures^22^. Studies on intraoperative fluid management are predominantly centered on patients undergoing intraabdominal surgeries, leaving a dearth of literature for head and neck procedures.

A central innovation of our study lies in the approach to pedicle catheterization. By establishing a novel animal model for free flap research, we hope to offer the opportunity of implementing randomized and standardized controlled trials to enhance the quality of research within this field. Unlike retrospective observational studies lacking randomization, standardization and reproducibility, this innovative study design holds the potential to generate high-quality evidence influencing clinical recommendations and hence improve patient care in perioperative settings following free flap reconstruction. While overhydration during head and neck cancer surgeries is linked to additional complications, inadequate resuscitation can potentially result in postoperative flap thrombosis^22,23^. Crystalloid volumes should not exceed 130 ml/kg in 24 h. Recent evidence supports the use of crystalloids over colloids in fluid resuscitation, as colloids are more expensive and may even increase mortality^24^. Blood transfusion in patients with head and neck free flap does not influence flap survival but is linked to elevated perioperative complications. A retrospective study demonstrated increased wound infection rates and mortality when accounting for factors such as age, preoperative hemoglobin, albumin, cancer stage and adverse pathological features. Recent findings suggest considering a restrictive transfusion approach in patients with free flap^25^.

### Free flap perfusion and metabolism

Any free flap transfer is associated with an unavoidable period of anoxia, resulting in varying degrees of tissue damage depending on the duration and type of tissue transplanted. Upon blood flow termination, tissue flaps enter a state of anoxia. Concomitantly, anaerobic metabolic activity results in the accumulation of lactate, a decline in intracellular pH, a reduction in adenosine triphosphate (ATP) levels, an increase in Ca^2+^ concentration and a buildup of pro-inflammatory mediators. The magnitude of injury attributable to primary ischemia shows a direct correlation with the duration of the ischemic event^26^. Restoration of blood flow (reperfusion) initiates the next step of tissue damage via reperfusion damage (ischemia and reperfusion (I/R) injury). I/R injury encompasses the activation of programmed cell death, endothelial dysfunction, transcriptional reprogramming and stimulation of both the innate and adaptive immune systems^27^. Free flaps result in denervation, variable primary ischemia and loss of sympathetic tone. Despite these changes, arteries and veins retain innervation, responding to local, physical and chemical stimuli. The absence of lymphatic drainage increases the risk of interstitial edema, with heightened sensitivity to fluid leakage and pressure. Blood flow is typically reduced to half of its original rate, requiring days or weeks to normalize.

Through continuous monitoring and comparison of venous blood gas parameters between the flap and central venous circulation, we have obtained specific information about the pathophysiological processes in free flaps following hemorrhagic shock with correspondingly different volume substitution schemes. During hemorrhagic shock, pO_2_^(cv-BGA)^ decreased significantly more compared to pO_2_^(flap-BGA)^. From t7 to t10, pO2^(flap-BGA)^ was significantly increased in the group with STERO substitution compared to STERO+, AUTO and GELA groups (Fig. 2a). The authors find no convincing rationale to explain why the administration of isotonic full-electrolyte solution should result in elevated pO2^(flap-BGA)^ compared to the other groups. This observation is most likely attributable to the limited sample size (*n* = 7), rather than any inherent property of the solution. By contrast, pCO_2_ increased in the central venous blood during the shock phase above the upper reference range and subsequently decreased until t10, with no discernible difference between groups. The average values of pO_2_ and pCO_2_ in the present study are consistent with those found in other investigations^28–30^. Studies involving both animal models and human participants have revealed a substantial dissociation between arterial and venous pCO_2_ in situations where oxygen delivery is decreased due to reduced cardiac output, such as hemorrhagic shock^31,32^ or septic shock^33^. In instances of hemodynamic compromise, as observed in cases with major blood loss, carbon dioxide accumulates quickly, before the identification of substantial levels of organic acids in the bloodstream caused by the healthy liver’s ability to increase lactate metabolism during the initial phase of hemorrhage^34^.

The cv-BGA baseline values for pH and lactate are comparable to those reported in the relevant literature^30^. However, the pH value in the venous blood of the transplant demonstrates a less pronounced decline during shock (t5). Post-resuscitation and up to 4 h later (t10), pH levels consistently rose within the physiological reference range without differences among substitution groups. Concurrently, a significant increase in lactate was observed in the flap’s venous BGA after reperfusion (t4) and during shock (t5) compared to central venous blood. Lactate levels later decreased but remained above the reference range in the flap, irrespective of group, even 4 h after reperfusion. As oxygen supply decreases, cells shift to anaerobic metabolism, reducing ATP production and causing intracellular acidosis from lactate formation. This phenomenon leads to lysosomal membrane destabilization, enzyme leakage, cytoskeleton breakdown and Na^+^/K^+^ ATPase activity inhibition^35^. The final process results in intracellular Na^+^ ions and water buildup, causing cellular swelling. Decreased Ca^2+^ excretion leads to intracellular Ca^2+^ accumulation, activating Ca^2+^-dependent proteases such as calpains^35,36^. The aforementioned processes are evident in the venous BGA results from our study showing significantly increased K^+^ and Cl^−^ levels, as well as decreased Na^+^ and Ca^2+^ levels in flap-BGA compared to cv-BGA following I/R. These observed changes in electrolyte levels are consistent with the physiological processes described. During shock and reduced oxygen supply, ATP depletion inhibits the Na^+^/K^+^ ATPase pump, resulting in intracellular accumulation of Na^+^ and cellular loss of K^+^, which explains the increase in extracellular K^+^ levels observed in the flap’s venous blood. The reduction in ATP also impairs Ca^2+^ pumps, decreasing the cell’s ability to expel Ca^2+^ and leading to intracellular Ca^2+^ accumulation. This activates Ca^2+^-dependent proteases such as calpains, which degrade cellular structures. Physiologically, Cl^−^ ions follow Na^+^ into the cell to maintain electrical neutrality, leading to intracellular rather than extracellular Cl^−^ accumulation. The initial (t4) increase of Cl^− flap-BGA^ compared to Cl^− cv-BGA^ observed in this study can be attributed to cellular damage with subsequent release of intracellular contents. During reperfusion, oxygen and pH levels return to normal, which is harmful to previously ischemic cells. Increased intracellular Ca^2+^ levels activate calpains, leading to cell damage and death^27^. Normoxemia restoration causes a surge in reactive oxygen species (ROS) production and a decrease in antioxidant capacity. Chouchani et al. found that ROS production is due to the reverse action of complex I of the electron transport chain, driven by accumulated succinate during ischemia^37^. The resulting ROS activate harmful pathways, causing cell membrane, cytoskeleton and DNA damage, disrupting ATP generation and inducing mitochondrial permeability transition pore^38^.

Furthermore, the combination of ROS, mitochondrial dysfunction and increased mitochondrial Ca^2+^ load opens the mitochondrial permeability transition pore, releasing substances such as cytochrome *c*, succinate and mitochondrial DNA. These substances can trigger cell death through apoptosis and necrosis and activate the immune system as danger/damage-associated molecular patterns^39^. I/R at the vascular level causes endothelial cell swelling, glycocalyx loss and cytoskeleton degradation, which increases vascular permeability and fluid loss^40^ This happens together with vasoconstriction due to the production of vasoactive substances such as endothelin 1 or platelet-derived growth factor and decreased nitric oxide production caused by decreased expression of endothelial nitric oxide synthase^35,41,42^. Overall, the cellular response to oxygen depletion is a well-described principle with extensive investigation of underlying pathways, including signaling factors, activation of anaerobic metabolism and ROS generation.

I/R injury further leads to leukocyte chemotaxis, endothelial adhesion and transmigration into the interstitial compartment. This process begins with increased P-selectin expression on endothelial cells, which interacts with P-selectin glycoprotein 1 on leukocytes. This interaction leads to the rolling of leukocytes on the endothelium^43^. Once activated, leukocytes release toxic substances that lead to further injury, including increased vascular permeability, edema, thrombosis and parenchymal cell death^35^. During I/R, 75% of platelets adhere to leukocytes attached to endothelial cells, while the rest of the platelets bind directly to the endothelium. This process allows for substantial crosstalk between the three different cell types, influencing adhesive-dependent inflammatory and thrombogenic events. Activated platelets release various pro-inflammatory, mitogenic, cytotoxic and proapoptotic molecules, as well as microvesicles. These changes may contribute to platelet involvement in I/R-induced endothelial apoptosis and NETosis (activation and release of neutrophil extracellular traps). Platelet-induced promotion of leukocyte activation and adhesion has a critical role in post-ischemic tissue injury^35^.

The importance of ischemia time as a critical determinant of the above-mentioned mechanisms must be considered, representing a possible limitation of the presented study. Due to the limitation in sample size, a consistent duration of ischemia was maintained across experimental groups to avoid potential confounding factors and reduction of statistical power. However, this restriction in ischemic time limits the ability to fully explore the impact of varying ischemia times on flap metabolism. Further investigations with larger sample sizes should consider varying the ischemic period to provide a more comprehensive understanding of the influence of ischemia on flap metabolism and free flap outcomes.

Beyond the alterations in electrolytes, our study revealed a characteristic increase in lactate associated with ischemia. Lactate, more than just a metabolic byproduct, can influence the immune response. Depending on the specific cell type affected, it can either mitigate or exacerbate inflammation. Based on a systematic literature review, it has been shown that lactate induces NETosis in neutrophil granulocytes, which may subsequently lead to platelet activation and potential thromboembolic complications^44,45^. Within this context, it is important to acknowledge the association between various neoplasms, such as squamous cell carcinoma of the head and neck, and elevated neutrophil granulocyte levels, as well as cell-free DNA release via NETosis. This has been correlated with an increased risk of thromboembolic events. Given that free flap transfers in the head and neck region are primarily employed for defect reconstruction following ablative tumor resection, these factors may contribute to flap perfusion complications.

## Conclusion

In the context of managing volume for hemorrhagic shock, it is imperative to assess the effects on acid–base balance during free flap transfer. The use of crystalloid solutions supplemented with norepinephrine has been found to slightly disrupt this balance, particularly by elevating lactate levels. Contrary to the widely held belief that vasoactive agents impair free flap perfusion, our research indicates that, within the scope of volume management for hemorrhagic shock, these agents do not negatively impact free flap perfusion in the short term.

## Methods

### Institutional review board statement

The animal study protocol was approved by the institutional review board (or ethics committee) of Landesuntersuchungsamt Rheinland-Pfalz (approval no. G21-1-080; date of approval: 22 November 2021).

### Animals

All experiments were performed according to the German Animal Protection Law and the ARRIVE guidelines between January and September 2021. The trial was planned as a prospective, randomized trial. Thirty-seven German domestic pigs (*Sus scrofa* *domestica*; age 12–16 weeks, weight 29–34 kg) were included in the study, but 6 died early; thus, 31 animals were examined. Animals were starved 6 h before the experiment for minimized risk of aspiration during intubation, but water was always accessible ad libitum. To reduce stress, animals were maintained in their familiar environment as long as possible and were sedated with an intramuscular injection of azaperone (4 mg/kg) + ketamine (4 mg/kg) into the neck or gluteal muscle. The transport was supervised and accompanied by a veterinarian with continuous monitoring of peripheral oxygen saturation. Sedation, transport and instrumentation were performed as described in detail before^46^. General aaesthesia was induced by intravenous (ear) injection of fentanyl (4 µg/kg), propofol (4 mg/kg) and atracurium (0.5 mg/kg) and sustained during the whole experiment by continuous infusion of propofol (8–12 mg/kg/h) and fentanyl (0.1–0.2 µg/kg/h), whereby endotracheal intubation was conducted using a common endotracheal tube (6.0 mm) with volume-controlled ventilation (Evita 2, Draeger) positive end-expiratory pressure of 5 cmH_2_O; tidal volume of 8 ml/kg; fraction of inspired oxygen of 0.4; inspiration to expiration ratio of 1:2; and variable respiration rate to achieve end-tidal CO_2_ <6 kPa. Instrumentation was performed by placing intravenous sheaths in the femoral artery and vein under sonographic guidance on the left side.

### Flap preparation

In a previous study by our research group, we provided a detailed description of the flap preparation, catheterization of the venous flap pedicle and procedure for blood and tissue sampling^46^. In short, animals were placed in supine position with the right upper and lower limbs fully abducted and secured by bandages with anatomical landmarks drawn in projection on the skin (Supplementary Fig. 2). Surgery was performed using basic surgical instruments, monopolar needle and bipolar forceps, as well as microsurgical equipment (KLS Martin, Gebrüder Martin GmbH & Co. KG). Surgeons used 3.0× optical magnification (starMed GmbH & Co. KG) for flap preparation and 5.4× magnification for vascular anastomosis.

### Blood sampling and hyperspectral flap monitoring

Hyperspectral flap perfusion monitoring was performed preoperatively after dermal marking of anatomic landmarks to determine baseline tissue perfusion (t1). Subsequent measurements were made after flap preparation and before disconnection (t2), after arterial anastomosis but before flap reperfusion (t3), after flap reperfusion (t4), during hemorrhagic shock (t5), following circulatory stabilization (t6) and 1 h (t7), 2 h (t8), 3 h (t9) and 4 h (t10) after. Blood samples for venous BGA were regularly collected at the aforementioned time points from t4 to t10, from the flaps’ pedicle as well as from the central venous circulation.

### HSI

In this study, a hyperspectral sensor system (TIVITA Tissue System, Diaspective Vision GmbH) was used. The HSI sensor generates a three-dimensional data cube containing spatial information within the first two dimensions (resolution 0.1 mm per pixel at 50 cm distance) while the third dimension includes spectral information (resolution 5 nm). The method includes conventional as well as spectroscopic approaches to capture both spatial and spectral information of an image scene. While conventional RGB methods (red, green and blue) cover a limited wavelength spectrum visible to the human eye, HSI is able to process an electromagnetic wavelength spectrum >740 nm (ref. ^47^). Briefly, HSI is based on the assessment of contiguous spectra (that is, light of different wavelengths) individually re-emitted by molecules. These physicochemical raw data are then processed by computerized algorithms, specific for the respective molecule of interest (hyperspectral signatures), particularly hemoglobin, oxygenized hemoglobin and water^48,49^. Following HS image recording over 10 s, an additional 8 s are needed to compute a RGD (red, green and blue) true-color image and an additional four pseudo-color images, representing the parameters: StO_2_ (0–100%), NPI (as arbitrary units 0–100) and distribution of hemoglobin (THI as arbitrary units 0–100) and water (TWI as arbitrary units 0–100)^9,50^. Hemoglobin and its differentiation between its oxygenated and deoxygenated form has a central role in HSI perfusion monitoring^48^. Due to the high absorbance of hemoglobin between 570 and 590 nm, electromagnetic radiation with shorter wavelengths exhibits reduced tissue penetration. As a result, microcirculation can be detected up to a depth of 1 mm. StO_2_ evaluates the oxygen saturation in the microcirculation of surface tissue layers, encompassing both arterial and venous blood. This measurement directly showcases changes in oxygen provision and uptake in the tissue. Thus, StO_2_ primarily signifies the oxygen saturation from the venous segment (75%) of the microcirculation after the oxygen has been delivered to the tissue. The StO_2_ values of healthy volunteers are between 50% and 70% (ref. ^51^). There are no thresholds, but corresponding studies are currently being conducted. NPI describes the quality of blood flow, which is determined by the relative oxygen saturation of the hemoglobin and the relative hemoglobin content in deep tissue layers (4–6 mm)^9^. In the manufacturer’s software, parameters are displayed in false colors from red (high), through yellow and green, to blue (low). THI describes the relative amount of hemoglobin in the microcircular system of the tissue area under consideration. This parameter gives an indication of inflow and or outflow problems. TWI describes the relative water content in the assessed region of interest. We have described the importance of the parameters and their combination for perfusion assessment in detail in a previous paper^9^.

### Volume management and hemorrhagic shock

From baseline (t0) to the point of free flap transfer (t4), animals received an average fluid volume of 1,125 ml, utilizing a crystalloid solution. During the shock phase (t5) to the point of achieving circulatory stabilization (t6), the fluid volume administered, varying according to group allocation (STERO, 1,588 ml; STERO+, 1,755 ml; AUTO, 1,171 ml; or GELA, 1,450 ml), averaged 1,491 ml per animal. The total volume administered from t7 to t10 amounted to 2,414 ml, leading to an overall average volume of 5,030 ml per animal administered from the start (t0) to the end (t10) of the experiment. Flap reperfusion was followed by induction of hemorrhagic shock (60% drop in cardiac output and mean arterial blood pressure) with maintenance for 30 min and subsequent circulatory stabilization with (1) crystalloid solution (Sterofundin ISO, B. Braun SE), (2) crystalloid solution + catecholamine (Sterofundin ISO + norepinephrine), (3) autotransfusion (whole blood) and (4) colloidal solution (Gelafundin ISO 4%, B. Braun SE).

### Statistics

The raw data sets were stored in Excel sheets (Microsoft Corporation) and then imported into SPSS Statistics (version 23.0.0.2, MacOS X; SPSS Inc., IBM Corporation). The data are presented as mean ± standard deviation, minimum and maximum. The normal distribution of the data was checked using nonparametric Shapiro–Wilk and Kolmogorov–Smirnov tests. In addition, dependency analysis was conducted to identify or exclude differences and correlations. Statistical significance was assessed using ANOVA with LSD post-hoc test and Wilcoxon signed-ranks test (two-sided). Significant *P* values were considered as ≤0.05. Line charts with plotted mean ± s.d. were used for illustration. Due to a small number of similar studies and the absence of cut-off values, sample size estimation could not be conducted^52,53^.

### Reporting summary

Further information on research design is available in the Nature Portfolio Reporting Summary linked to this article.

## Online content

Any methods, additional references, Nature Portfolio reporting summaries, source data, extended data, supplementary information, acknowledgements, peer review information; details of author contributions and competing interests; and statements of data and code availability are available at 10.1038/s41684-024-01410-6.

## Supplementary information

Supplementary Information Supplementary Figs. 1 and 2.

Reporting Summary

## Data Availability

The data sets generated during and/or analyzed during the current study are available from the corresponding author on reasonable request.
