# Supplementary material for: Effects of volume management on free flap perfusion and metabolism in a large animal model study
Source: Lab Anim (NY). 2024 Aug 9;53(10):268–75. doi: 10.1038/s41684-024-01410-6 (PMC11439732; doi:10.1038/s41684-024-01410-6)
Supplement: Supplementary file 1 — Supplementary Figs. 1 and 2. [file 41684_2024_1410_MOESM1_ESM.pdf]

lab animal

Supplementary information

https://doi.org/10.1038/s41684-024-01410-6

Effects of volume management on free flap perfusion and metabolism in a large animal model study

In the format provided by the authors and unedited

Supplementary Information

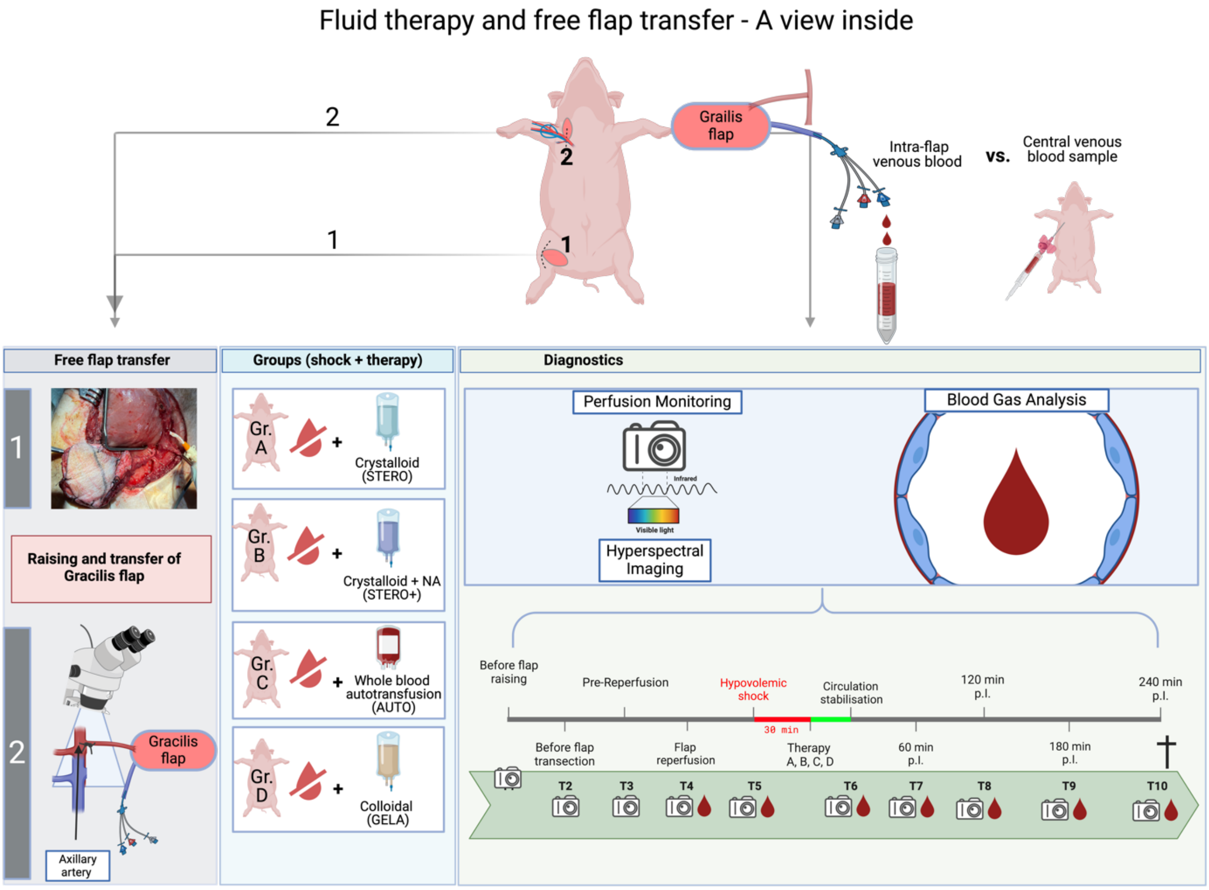

Supplementary Figure 1: Graphical abstract shows the experimental setup from lifting and transfer of the free gracilis graft, through the four different experimental groups A-D, as well as the diagnostic data acquisition and the corresponding measurement time points from T1-T10. Created with BioRender.com.

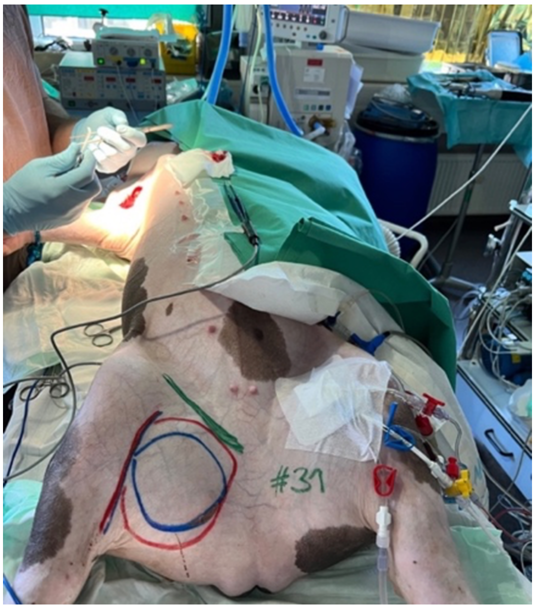

Supplementary Figure 2: Animal in supine position with abducted hind and forelimbs and marked anatomical landmarks. The inguinal ligament is marked in green, the extent of the skin island in blue, and the outline of the gracilis muscle in red. The vertical blue and red lines indicate the course of the palpable superficial femoral artery and vein. In the background, the preparation of the recipient vessel in the axillary region can be observed.
