# Supplementary material for: Effects of volume management on free flap perfusion and metabolism in a large animal model study
Source: Lab Anim (NY). 2024 Aug 9;53(10):268–75. doi: 10.1038/s41684-024-01410-6 (PMC11439732; doi:10.1038/s41684-024-01410-6)
Supplement: Supplementary file 2 — Reporting Summary [file 41684_2024_1410_MOESM2_ESM.pdf]

nature portfolio

Corresponding author(s): DBPR, LABAN-A01881-T

Last updated by author(s): 25.02.2024

Reporting Summary

Nature Portfolio wishes to improve the reproducibility of the work that we publish. This form provides structure for consistency and transparency in reporting. For further information on Nature Portfolio policies, see our Editorial Policies and the Editorial Policy Checklist.

Statistics

For all statistical analyses, confirm that the following items are present in the figure legend, table legend, main text, or Methods section.

n/a	Confirmed	
☐	☒	The exact sample size (*n*) for each experimental group/condition, given as a discrete number and unit of measurement
☐	☒	A statement on whether measurements were taken from distinct samples or whether the same sample was measured repeatedly
☐	☒	The statistical test(s) used AND whether they are one- or two-sided<br>*Only common tests should be described solely by name; describe more complex techniques in the Methods section.*
☐	☒	A description of all covariates tested
☐	☒	A description of any assumptions or corrections, such as tests of normality and adjustment for multiple comparisons
☐	☒	A full description of the statistical parameters including central tendency (e.g. means) or other basic estimates (e.g. regression coefficient) AND variation (e.g. standard deviation) or associated estimates of uncertainty (e.g. confidence intervals)
☐	☐	For null hypothesis testing, the test statistic (e.g. *F*, *t*, *r*) with confidence intervals, effect sizes, degrees of freedom and *P* value noted<br>*Give P values as exact values whenever suitable.*
☒	☐	For Bayesian analysis, information on the choice of priors and Markov chain Monte Carlo settings
☒	☐	For hierarchical and complex designs, identification of the appropriate level for tests and full reporting of outcomes
☒	☐	Estimates of effect sizes (e.g. Cohen's *d*, Pearson's *r*), indicating how they were calculated

Our web collection on statistics for biologists contains articles on many of the points above.

Software and code

Policy information about availability of computer code

Data collection	Raw data sets were stored in Excel® sheets (Microsoft Corporation, Redmond, USA)
Data analysis	Data were imported into SPSS Statistics® (version 23.0.0.2, MacOS X; SPSS Inc., IBM Corporation, Armonk, NY, USA) for statistical analysis.

For manuscripts utilizing custom algorithms or software that are central to the research but not yet described in published literature, software must be made available to editors and reviewers. We strongly encourage code deposition in a community repository (e.g. GitHub). See the Nature Portfolio guidelines for submitting code & software for further information.

Data

Policy information about availability of data

All manuscripts must include a data availability statement. This statement should provide the following information, where applicable:

- Accession codes, unique identifiers, or web links for publicly available datasets
- A description of any restrictions on data availability
- For clinical datasets or third party data, please ensure that the statement adheres to our policy

All raw data on which this study is based will be made available by the corresponding author upon request.

Human research participants

Policy information about studies involving human research participants and Sex and Gender in Research.

Reporting on sex and gender	*Use the terms sex (biological attribute) and gender (shaped by social and cultural circumstances) carefully in order to avoid confusing both terms. Indicate if findings apply to only one sex or gender; describe whether sex and gender were considered in study design whether sex and/or gender was determined based on self-reporting or assigned and methods used. Provide in the source data disaggregated sex and gender data where this information has been collected, and consent has been obtained for sharing of individual-level data; provide overall numbers in this Reporting Summary. Please state if this information has not been collected. Report sex- and gender-based analyses where performed, justify reasons for lack of sex- and gender-based analysis.*
Population characteristics	*Describe the covariate-relevant population characteristics of the human research participants (e.g. age, genotypic information, past and current diagnosis and treatment categories). If you filled out the behavioural & social sciences study design questions and have nothing to add here, write "See above."*
Recruitment	*Describe how participants were recruited. Outline any potential self-selection bias or other biases that may be present and how these are likely to impact results.*
Ethics oversight	*Identify the organization(s) that approved the study protocol.*

Note that full information on the approval of the study protocol must also be provided in the manuscript.

Field-specific reporting

Please select the one below that is the best fit for your research. If you are not sure, read the appropriate sections before making your selection.

☒ Life sciences ☐ Behavioural & social sciences ☐ Ecological, evolutionary & environmental sciences

For a reference copy of the document with all sections, see nature.com/documents/nr-reporting-summary-flat.pdf

Life sciences study design

All studies must disclose on these points even when the disclosure is negative.

Sample size	Due to the lack of corresponding preliminary data, the above study was conducted purely as an orientation study with explorative-descriptive statistics, whereby a large number of established parameters were collected. Preliminary case number planning was therefore only possible to a limited extent for this study. In prior studies by our group, various questions were investigated in the animal model. Here, group sizes of n=8 proved to be adequate to demonstrate significant differences due to treatment despite individual fluctuations in animals and shock induction. This was therefore taken as the basis for the case number planning.
Data exclusions	A total of 37 animals were used, of which five animals died early due to cardiac arrhythmia and had to be excluded.
Replication	We have undertaken several measures to ensure the reliability and repeatability of our results. Firstly, all experiments were conducted at least 8 times (8 animals per group) to account for biological variability and to ensure statistical robustness. We also employed standardized protocols and materials across all experimental runs to minimize variation. To enhance the reproducibility of our findings, a separate team within our laboratory independently repeated a subset of the experiments. This approach ensured that the results were not influenced by individual biases, confirming the integrity of our findings through successful replication across all attempts. Consistent outcomes across these experiments further attest to the robustness of our results. Although any non-replicable or irreproducible findings would have been rigorously analyzed and reported, highlighting potential variables such as experimental conditions or sample variability, we are pleased to report that all efforts to replicate our experiments corroborated the original results, evidencing a high level of reproducibility.<br>Exception: From t7 to t10, pO2flap-BGA was significantly increased in the group with STERO substitution compared to STERO+, AUTO, and GELA ($p < 0.05$, Fig. 4A). The authors find no convincing rationale to explain why the administration of isotonic full-electrolyte solution should result in elevated pO2flap-BGA compared to the other groups. This observation is most likely attributable to the limited sample size, with n = 7, rather than any inherent property of the solution
Randomization	The assignment is made by blindly pulling a sealed envelope with a group as its contents.
Blinding	Blinding was not possible after group allocation due to the methodological protocol (e.g. autotransfusion versus Gelafundin). Furthermore, it would have had no influence on the conduct of the trial or the acquisition of data.

Reporting for specific materials, systems and methods

We require information from authors about some types of materials, experimental systems and methods used in many studies. Here, indicate whether each material, system or method listed is relevant to your study. If you are not sure if a list item applies to your research, read the appropriate section before selecting a response.

Materials & experimental systems

n/a	Involved in the study
☒	☐ Antibodies
☒	☐ Eukaryotic cell lines
☒	☐ Palaeontology and archaeology
☐	☒ Animals and other organisms
☒	☐ Clinical data
☒	☐ Dual use research of concern

Methods

n/a	Involved in the study
☒	☐ ChIP-seq
☒	☐ Flow cytometry
☒	☐ MRI-based neuroimaging

Animals and other research organisms

Policy information about studies involving animals; ARRIVE guidelines recommended for reporting animal research, and Sex and Gender in Research

Laboratory animals	No laboratory animals used.
Wild animals	Thirty-two German domestic pigs (sus scrofa domestica; age: 12–16 weeks, weight: 29–34 kg) were examined. Animals were starved six hours prior to the experiment for minimized risk of aspiration during intubation but water was accessible ad libitum at all times. In order to reduce stress animals maintained in their familiar environment as long as possible and were sedated with an intramuscular (i.m.) injection of Azaperone (4mg/kg) + Ketamine (4mg/kg) into the neck or gluteal muscle. The transport was supervised and accompanied by a veterinarian with continuous monitoring of peripheral oxygen saturation. The experiment was a final experiment in which the animals were not awakened from anesthesia, but were euthanized by controlled bleed out and intravenous potassium injection.
Reporting on sex	Only male animals are used for the experimental project. These are not subject to cyclical hormone release and are therefore easier to compare with each other, as otherwise an uncontrollable factor would be added, which would significantly increase the number of test animals.
Field-collected samples	Study did not involve sample collection from the field
Ethics oversight	This animal trial was approved by the State and Institutional Animal Care Committee Rhineland Palatine (approval no. G21-1-080), and all experiments were performed according to the German Animal Protection Law and the ARRIVE guidelines between January and September 2021.

Note that full information on the approval of the study protocol must also be provided in the manuscript.
